# Cluster B personality disorders and psychotropic medications: a focused analysis of trends and patterns across sex and age groups

**DOI:** 10.1007/s00127-024-02768-1

**Published:** 2024-09-17

**Authors:** Carlotta Lunghi, Lionel Cailhol, Victoria Massamba, Suzane Renaud, Pierre David, Elhadji A. Laouan Sidi, Robert Biskin, Marion Koch, Cathy Martineau, Elham Rahme, Louis Rochette, Caroline Sirois, Evens Villeneuve, Philippe Vincent, Alain Lesage

**Affiliations:** 1 Department of Medical and Surgical Sciences, Alma Mater University of Bologna, Via Irnerio, 48, 40126 Bologna, Italy; 2 Institut National de Santé Publique du Québec, Quebec, QC Canada; 3 Department of Psychiatry and Research Center, Institut Universitaire de Santé Mentale de Montréal, Montreal, QC Canada; 4 Department of Psychiatry and Addiction, Université de Montréal, Montreal, QC Canada; 5 Department of Psychiatry, Centre Intégré de Santé et de Services Sociaux (CISSS) des Laurentides, Saint-Jérôme, QC Canada; 6 Department of Psychiatry, McGill University, Montreal, QC Canada; 7 Department of Psychiatry, Hôpital de Gatineau, Gatineau, QC Canada; 8 Department of Health Sciences, Université du Québec à Rimouski, Lévis, QC Canada; 9 Department of Medicine, Division of Clinical Epidemiology, McGill University, Montreal, QC Canada; 10 Faculty of Pharmacy, Université Laval, Quebec, QC Canada; 11 Faculty of Medicine, Université Laval, Quebec, QC Canada; 12 Institut Universitaire en Santé Mentale de Québec, Quebec, QC Canada; 13 Faculty of Pharmacy, Université de Montréal, Montreal, QC Canada

**Keywords:** Personality disorders, Psychotropic medications, Sex differences, Age differences, Prescription patterns, Drug utilization research

## Abstract

**Purpose:**

This study investigated sex and age differences in patterns of psychotropic medication use before and after the initial diagnosis of Cluster B personality disorders (PDs) and analyzed trends over time.

**Methods:**

Analyzing data from the Quebec Integrated Chronic Disease Surveillance System for individuals newly diagnosed with Cluster B PD (≥ 14 years) between 2002 and 2018 and under the provincial public drug plan, we calculated yearly and monthly proportions of individuals exposed to psychotropic medications during the year before and after their diagnosis by sex and age. Robust Poisson regression models assessed the association between sex and exposure to psychotropic medications after the diagnosis of Cluster B PD.

**Results:**

Among 87,778 individuals with a first Cluster B PD diagnosis (mean age: 44.5 years; 57.5% women), the proportion of users increased post-diagnosis. Notably, after diagnosis, females were more likely to receive psychiatric medications (between 78.9% and 83.7% during the study period vs. 72.8% and 76.8%). Males were less likely than females to receive antidepressants (adjusted prevalence ratio (aPR): 0.83; 99% confidence interval (CI): 0.82–0.85) and anxiolytics (aPR: 0.86; 99%CI: 0.84–0.88), whereas they had higher exposure to antipsychotics (aPR: 1.04; 99%CI: 1.02–1.06) and ADHD medications (aPR: 1.14; 99%CI: 1.07–1.2). Age-specific trends showed increased ADHD medication use among younger patients (14–24 years), and anxiolytic use predominated in those aged **≥** 65 years.

**Conclusions:**

Psychotropic medication use was high among Cluster B PD patients, with differences in medication classes according to age and sex. The marked sex and age differences in psychotropic medication use among Cluster B PD patients underscore the need for a sex-sensitive and age-specific approach in psychiatric care.

**Supplementary Information:**

The online version contains supplementary material available at 10.1007/s00127-024-02768-1.

## Introduction

Cluster B personality disorders (PDs) represent a significant public health concern, characterized by their complex, severe manifestations such as dramatic, emotional, and erratic behaviours [1]. The Diagnostic and Statistical Manual of Mental Disorders, Fifth Edition (DSM-5) includes four types of Cluster B PDs: antisocial (ASPD), borderline (BPD), histrionic (HPD), and narcissistic (NPD) [1]. Estimates place the lifetime prevalence of Cluster B PDs at 2.6% in Quebec, Canada, in 2011–2012 [2], underscoring regional variations in prevalence rates compared to the higher estimate of 5.5% found in Western countries, with differences depending on subtype (0.8% for HPD, 1.2% for NPD, 1.9% for BPD, and 3.1% for ASPD) [3]. Many studies reported higher prevalence rates of Cluster B PDs in women than in men, especially BPD and HPD [5]. Nonetheless, other studies suggest similar prevalence rates of BPD between men and women [8, 9]. Conversely, ASPD is more frequently diagnosed in men, a trend that may reflect not only inherent gender differences but also potential biases in clinical practice, leading to overlooked diagnoses in men [7]. However, the differential sex prevalence of PDs in clinical settings may be largely due to sampling bias [10] or a delayed diagnosis in men. Indeed, it has been reported that men with BPD are diagnosed on average four years later than women [10]. Cluster B PDs are typically diagnosed in late adolescence or early adulthood, but their symptoms may persist across the lifespan [11, 12]. Nevertheless, their prevalence seems to decrease with age. Clinically, different features of these disorders have been reported, with differences possibly due to diagnostic criteria focusing on behavioural acting out, which is more prevalent in youth and tends to lessen with maturity [11, 13, 14].

Addressing Cluster B PD symptomatology remains a significant clinical challenge [15], with psychotherapy being a critical, evidence-based cornerstone of a comprehensive treatment plan [16]. Although there are no medications specifically approved for PDs, pharmacotherapy can have a pivotal role in managing the comorbid conditions often accompanying these disorders, such as impulsivity and anxious-depressive symptoms, underscoring the complexity of treatment strategies [14, 17–19]. Moreover, psychotropic medications may also be prescribed when psychotherapy is inaccessible [20]. Research suggests that there may be sex differences in medication use among patients with Cluster B PDs, which could be due to differences in the features and expression of PDs between the sexes. This could be because of the differences in the symptoms and manifestation of PDs between men and women [21–23], Men tend to have externalizing symptoms, while women are more likely to have symptoms of anxiety and depression [21]. Recent research found that women diagnosed with BPD were more inclined to take psychotropic medications, particularly sedatives and antidepressants, while men were more likely to use anti-addiction drugs [24].

To the best of our knowledge, there is no published study focussing on differences and trends in psychopharmacological treatment across sex and age. Thus, this study aims to investigate psychotropic medication use variations across different age and sex groups before and after the diagnosis of Cluster B PDs and identify trends or patterns over time, addressing a critical gap in the literature and aiming to inform more nuanced, equitable treatment approaches.

## Methods

### Data source

Using the Quebec Integrated Chronic Disease Surveillance System (QICDSS) database [25], we conducted a population-based cohort study of Quebec residents covered under the universal provincial healthcare program. The QICDSS contains information on diagnoses from physician visits claims and inpatient discharge summaries based on the ninth and tenth revisions of the International Classification of Diseases (ICD-9 and ICD-10) starting from January 1, 1996, along with a linkage to the death registry. In addition to the above, the QICDSS database includes sociodemographic information and dispensing (claim) data on prescribed medications that are covered by the public drug insurance plan for residents without a private plan, those who receive a guaranteed income supplement or welfare, and all citizens who are 65 years old and above and do not reside in long-term care facilities. The provincial public drug plans cover almost 45% of Quebec citizens [26].

### Population

We retrieved data from the QICDSS database between April 1, 2002, and March 31, 2019, to find all Quebec residents who were 14 years or older and had been diagnosed for the first time with a Cluster B PD (with a minimum 5-year period to identify the first diagnosis registered in the database). To be considered as having Cluster B PD, individuals had to have received at least one ICD-9 or ICD-10 diagnostic code during a physician visit or hospitalization, according to a previously described case definition, elaborated by a group of psychiatrists and psychologists specialized in treating PDs in Quebec [2]. Briefly, a list of ICD-9 and ICD-10 codes (301.1, 301.3, 301.5, 301.7, 301.8, 301.9, and F070, F340, F341, F488, F602, F603, F604, F606, F608, F609, F61, F620, F621, F628, F629, F681, F688, F69) was selected to identify core symptoms of Cluster B PDs according to the DSM, as no code specifically identify Cluster B PD in ICD. To gather data on prescribed medication, we only included patients covered by the provincial public drug plan within a two-year window surrounding their first diagnosis of Cluster B PD (cohort entry). We had to exclude those with private medical insurance because information on their prescribed drugs is not available in the QICDSS database.

### Medication use

During the study period, we collected all dispensing claims for prescribed medications to identify the use of psychotropic medications. This was done for the years before and after cohort entry. The psychotropic medications were categorized into five main classes based on the American Hospital Formulary Service (AHFS) classification [27] and common drug denomination (chemical name of the medication). These classes included antipsychotics, antidepressants, anxiolytics, mood stabilizers, and ADHD medications (Supplemental Table 1).

### Sociodemographic and clinical information

At cohort entry, we gathered sociodemographic information included in the QICDSS database, such as biological sex at birth, age at the diagnosis (further classified as 14–24 years; 25–49 years; 50–64 years; 65 years and above), social and material deprivation (measured using an ecological index based on the census dissemination area approximating the individual’s socioeconomic status and reported as quintiles [28], and the area of residence (based on the Quebec census and classified as Montreal census metropolitan area (CMA), > 1,000,000 inhabitants; Other CMA, 100,000–1,000,000 inhabitants; Agglomerations, 10,000-100,000 inhabitants; and Small town/rural area, < 10,000 inhabitants).

Clinical information included the presence of psychiatric comorbidities (i.e., a claim for a physician visit or a hospitalization with a relevant ICD-9 or ICD-10 code) diagnosed in the five years before cohort entry according to previously described and validated case definitions [25].

### Statistical analyses

We used descriptive statistics to report baseline sociodemographic and clinical characteristics as proportions and 99% confidence intervals (CIs) for the study cohort separately for male and female individuals. We evaluated the number of psychotropic medications claimed within a year before and after each subject’s diagnosis of Cluster B PD, by identifying the chemical entity through common drug denominations. We then determined the yearly and monthly proportion of individuals exposed to these medications by psychotropic medication classes (antipsychotics, antidepressants, anxiolytics, mood stabilizers, and ADHD medications), according to sex and age groups (14–24 years; 25–49 years; 50–64 years; 65 years and above). Monthly granularity allowed for a detailed examination of trends and changes in medication use surrounding the diagnostic period, providing insights into the immediate impacts of a Cluster B PD diagnosis on treatment practices. Robust Poisson regression models were used to identify the association between sex and exposure to the five classes of psychiatric medication classes in the year after the diagnosis of Cluster B PD, adjusting for age, material and social deprivation, psychiatric comorbidities in the 5 years before cohort entry, and the year of PD diagnosis. We thus estimated unadjusted and adjusted prevalence ratios (PRs and aPRs, respectively) with their 99% CIs. All the analyses were performed using SAS Enterprise Guide 9.4.

## Results

We identified 87,778 individuals with a first diagnosis of Cluster B PD between 2002 and 2018 and full coverage under the provincial public drug plan in the two years within the cohort entry. Table 1 provides information on the sociodemographic and clinical characteristics of the cohort. Most individuals were female (57.5%), and the most represented age group was 25–49 years old. Females were more likely to have been prescribed antidepressants and anxiolytics in the year before the diagnosis of Cluster B PD, while males were more likely to have been prescribed antipsychotics and ADHD medications. The most common psychiatric conditions among both males and females were depression and anxiety disorders, affecting 55.5% and 54.3% of females and 44.6% and 43.4% of males, respectively. Most individuals had one or two recorded comorbidities in the QICDSS database.

**Table 1:** Sociodemographic and clinical characteristics individuals with a first diagnosis of cluster B personality disorder

Characteristics	Females (*N* = 50,431)		Males (*N* = 37,347)		All (*N* = 87,778)	
	*N* ^#^	% (99% CI)	*N* ^#^	% (99% CI)	*N* ^#^	% (99% CI)
**Age (years)**						
**14–24**	9850	19.5 (19.1–20.0)	6465	17.3 (16.8–17.8)	16,315	18.6 (18.3–18.9)
**25–49**	20,710	41.1 (40.5–41.6)	17,115	45.8 (45.2–46.5)	37,825	43.1 (42.7–43.5)
**50–64**	9795	19.4 (19.0-19.9)	7550	20.2 (19.7–20.8)	17,345	19.8 (19.4–20.1)
**65+**	10,070	20.0 (19.5–20.4)	6215	16.6 (16.2–17.1)	16,290	18.6 (18.2–18.9)
**Material deprivation** ^*^ **(quintile)**						
**1 (least deprived)**	6245	12.4 (12.0-12.8)	4565	12.2 (11.8–12.6)	10,805	12.3 (12.0-12.6)
**2**	7545	15.0 (14.5–15.4)	5520	14.8 (14.3–15.3)	13,065	14.9 (14.6–15.2)
**3**	9300	18.4 (18.0-18.9)	6830	18.3 (17.8–18.8)	16,130	18.4 (18.0-18.7)
**4**	11,020	21.8 (21.4–22.3)	8135	21.8 (21.2–22.3)	19,150	21.8 (21.5–22.2)
**5 (most deprived)**	12,885	25.6 (25.1–26.1)	9685	25.9 (25.4–26.5)	22,575	25.7 (25.3–26.1)
**Missing**	3435	6.8 (6.5–7.1)	2615	7.0 (6.7–7.3)	6050	6.9 (6.7–7.1)
**Social deprivation** ^*^ **(quintile)**						
**1 (least deprived)**	5345	10.6 (10.2–11.0)	4330	11.6 (11.2–12.0)	9675	11.0 (10.8–11.3)
**2**	6815	13.5 (13.1–13.9)	5220	14.0 (13.5–14.4)	12,040	13.7 (13.4–14.0)
**3**	8130	16.1 (15.7–16.5)	5975	16.0 (15.5–16.5)	14,105	16.1 (15.7–16.4)
**4**	10,870	21.6 (21.1–22.0)	7780	20.8 (20.3–21.4)	18,645	21.2 (20.9–21.6)
**5 (most deprived)**	15,835	31.4 (30.9–31.9)	11,430	30.6 (30.0-31.2)	27,265	31.1 (30.7–31.5)
**Missing**	3440	6.8 (6.5–7.1)	2615	7.0 (6.7–7.3)	6050	6.9 (6.7–7.1)
**Geographical area** ^**$**^						
**CMA Montréal**	22,355	44.3 (43.8–44.9)	16,110	43.1 (42.5–43.8)	38,470	43.8 (43.4–44.3)
**Other CMA**	10,565	21.0 (20.5–21.4)	7870	21.1 (20.5–21.6)	18,435	21.0 (20.6–21.4)
**Agglomerations**	7335	14.5 (14.1–14.9)	5205	13.9 (13.5–14.4)	12,540	14.3 (14.0-14.6)
**Small town/rural area**	9930	19.7 (19.2–20.1)	7850	21.0 (20.5–21.6)	17,780	20.3 (19.9–20.6)
**Missing**	240	0.5 (0.4–0.6)	310	0.8 (0.7–0.9)	550	0.6 (0.6–0.7)
**Psychiatric medications** ^**+**^						
**Antipsychotics**	21,925	43.5 (42.9–44.0)	17,710	47.4 (46.7–48.1)	39,635	45.2 (44.7–45.6)
**Antidepressants**	30,575	60.6 (60.1–61.2)	17,400	46.6 (45.9–47.2)	47,970	54.7 (54.2–55.1)
**Anxiolytics**	22,600	44.8 (44.3–45.4)	14,000	37.5 (36.8–38.1)	36,605	41.7 (41.3–42.1)
**Mood stabilizers**	8570	17.0 (16.6–17.4)	6355	17.0 (16.5–17.5)	14,925	17.0 (16.7–17.3)
**ADHD medications**	2800	5.6 (5.3–5.8)	2380	6.4 (6.0-6.7)	5175	5.9 (5.7–6.1)
**Psychiatric comorbidity** ^**&**^						
**Schizophrenia**	4025	8.0 (7.7–8.3)	5140	13.8 (13.3–14.2)	9165	10.4 (10.2–10.7)
**Other psychosis**	5090	10.1 (9.7–10.4)	5375	14.4 (13.9–14.9)	10,470	11.9(11.6–12.2)
**Depression**	27,970	55.5 (54.9–56.0)	16,665	44.6 (44.0-45.3)	44,630	50.8 (50.4–51.3)
**Bipolar disorders**	10,655	21.1 (20.7–21.6)	7205	19.3 (18.8–19.8)	17,855	20.3 (20.0-20.7)
**Anxiety disorders**	27,405	54.3 (53.8–54.9)	16,225	43.4 (42.8–44.1)	43,625	49.7 (49.3–50.1)
**Adaptive disorders**	14,245	28.2 (27.7–28.8)	8285	22.2 (21.6–22.7)	22,525	25.7 (25.3–26.0)
**Alcohol abuse disorders**	5460	10.8 (10.5–11.2)	6875	18.4 (17.9–18.9)	12,340	14.1 (13.8–14.4)
**Drug abuse disorders**	7050	14.0 (13.6–14.4)	7915	21.2 (20.6–21.7)	14,965	17.0 (16.7–17.4)
**ADHD**	1735	3.4 (3.2–3.6)	1735	4.6 (4.4–4.9)	3460	3.9 (3.8–4.1)
**Eating disorders**	260	0.5 (0.4–0.6)	30	0.1 (0.1–0.1)	295	0.3 (0.3–0.4)
**Number of psychiatric comorbidities** ^**&**^						
**0**	8635	17.1 (16.7–17.6)	8145	21.8 (21.3–22.4)	16,780	19.1 (18.8–19.5)
**1–2**	23,690	47.0 (46.4–47.5)	16,230	43.5 (42.8–44.1)	39,920	45.5 (45.0-45.9)
**3–4**	14,520	28.8 (28.3–29.3)	9515	25.5 (24.9–26.1)	24,035	27.4 (27.0-27.8)
**5–6**	3235	6.4 (6.1–6.7)	3015	8.1 (7.7–8.4)	6245	7.1 (6.9–7.3)
**7+**	350	0.7 (0.6–0.8)	440	1.2 (1.0-1.3)	795	0.9 (0.8-1.0)

ADHD: attention-deficit/hyperactivity disorder; CI: confidence interval; CMA: census metropolitan area; B-PD: Cluster B personality disorder

^#^randomly rounded to 0/5; B-PD ^*^Pampalon index at Cluster B personality disorder (PD) diagnosis; ^**$**^ Montreal CMA: Montreal and Laval; Other CMA: Quebec, Sherbrooke, Trois-Rivières, Saguenay and Gatineau; ^**+**^Psychiatric medications claimed in the year before Cluster B-PD diagnosis; ^&^Psychiatric comorbidities in the 5 years before Cluster B-PD diagnosis; ^+^Fiscal years are comprised between the 1st of April of one year and the 31st of March of the following year

**Fig. 1:**
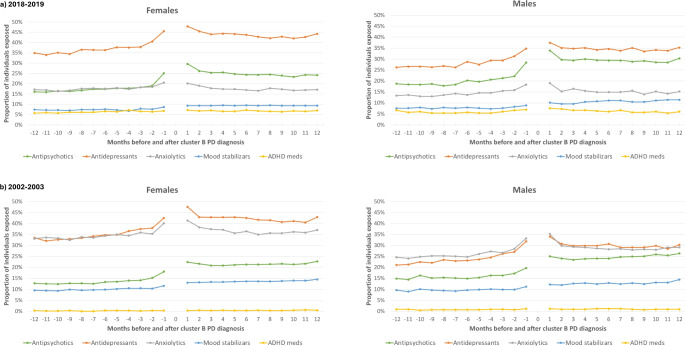
Proportion of individuals exposed to different classes of psychotropics in the 12 months before and after the Cluster B personality disorder diagnosis, by month, according to sex

Figure 1 presents the monthly proportions of individuals who used psychotropic medications in the year preceding and following their initial diagnosis of Cluster B PD, sorted by sex for the first (2002–2003) and the last (2018–2019) fiscal years. The figure indicates a general increase in medication usage for both males and females near the diagnosis, particularly for antipsychotics, antidepressants, and anxiolytics. In 2018–2019, while the usage proportions for anxiolytics, ADHD medication, and mood stabilizers (for females only) returned to pre-diagnostic values after the diagnosis, they remained high for antidepressants and antipsychotics for both sexes, with males showing a particularly high usage rate for antipsychotics.

For patients diagnosed during 2018–2019, in the year after diagnosis, the proportions of prescribed psychotropic medications across different classes varied between male and female patients. In the antipsychotic class, quetiapine was the predominant medication, comprising 54.2% of all such prescriptions for females and 47.5% for males, followed by risperidone (13.3% and 13.9%). The third most prescribed antipsychotic in females was aripiprazole (12.2%) and olanzapine (13.9%) in males. For antidepressants, the three most prescribed medications were venlafaxine (20.0% of all antidepressant prescriptions in females and 22.2% in males), citalopram (17.8% vs. 16.6%), and sertraline (14.8% vs. 13.2%). Similarly, for anxiolytics, clonazepam, lorazepam, and oxazepam were the three most frequently prescribed medications in this class for both female and male patients, accounting for, respectively, 34.5% vs. 39.7%, 29.7% vs. 27.3%, and 16.2% vs. 11.2%. Valproate was the leading mood stabilizer, representing 30.6% of mood stabilizer prescriptions in females and 34.9% in males. In females, lithium (15.1%) and topiramate (14.8%) followed, whereas in males, lamotrigine accounted for 17.6% of prescriptions in this class, followed by carbamazepine (15.3%). In the ADHD medication class, methylphenidate was the predominant prescription (45.6% of ADHD medication prescriptions in females and 40.3% in males), followed by lisdexamfetamine (41.0% in females vs. 33.4% in males) and atomoxetine (6.1% vs. 11.9%).

**Fig. 2:**
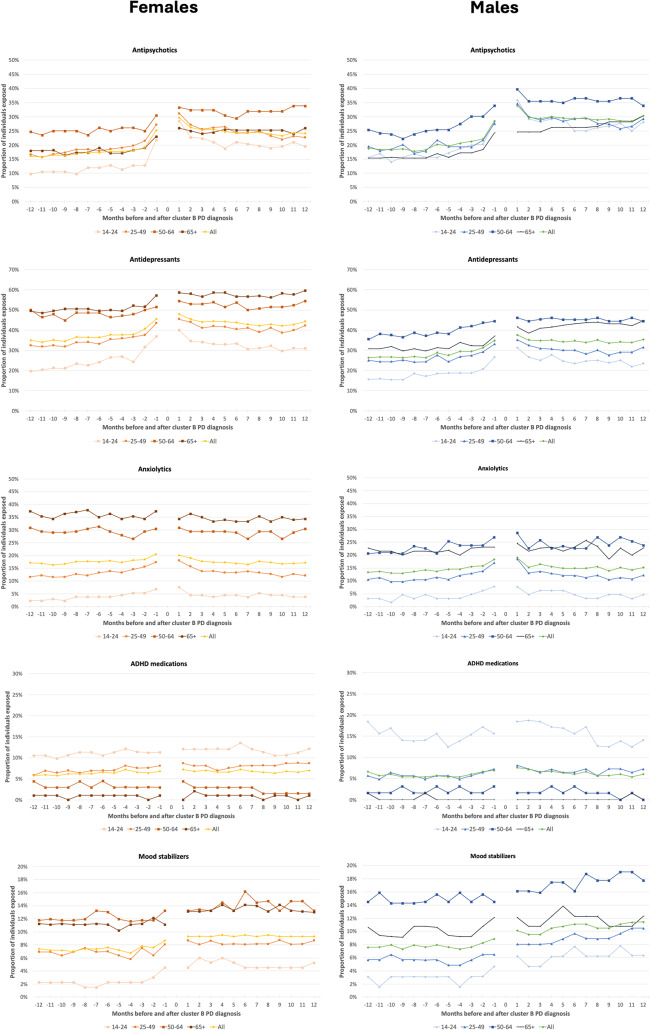
Proportion of individuals exposed to psychotropic classes in the 12 months before and after Cluster B PD diagnosis, by month, according to sex and age groups (2018–2019)

When examining monthly user proportions based on both sex and age groups, we could identify substantial differences (as shown in Fig. 2). For antipsychotics, the group with the higher proportion of users was that of 50 to 64 years, similarly for males and females. Nevertheless, there were differences depending on the sex, as it was observed that among females, the youngest group (14–24 years) had the lowest proportion of users. On the other hand, among males, the oldest group had the lowest proportion of users, with the youngest group following closely. Except for those aged 65 and above, a larger proportion of males rather than females used antipsychotics across all age groups, particularly in the year after being diagnosed with Cluster B PD. Notably, a significant proportion of antipsychotic prescriptions comprised low-dose quetiapine, as detailed in Supplemental Fig. 1. More females than males used antidepressants, independently of the age group, but especially those 65 years old or older, followed by those in the 50–64 age group. On the contrary, among males, those in the 50–64 age group had the highest proportion of users, although the difference with the other age groups generally decreases after being diagnosed with Cluster B PD. Anxiolytics showed similar patterns, with higher proportions of use among older individuals, especially among females, while the proportion of users did not differ much between males and females in the youngest groups. ADHD medications were used by the youngest individuals independently of the sex, with a particularly high proportion in males in the 14 to 24 years age group. Among them, the proportion of users a few months after the PD diagnosis decreased to a proportion lower than the year before. Finally, mood stabilizers were primarily used by older individuals independently of the sex, but with a particularly high proportion among males in the 50–64 age group, where the increase in use after the diagnosis was more evident and persisted one year later.

Table 2 reports the male vs. female PRs and aPRs of the Poisson regression models showing that, when adjusting for important sociodemographic and clinical variables, males were a little bit more likely to receive treatments with antipsychotics (aPR: 1.04; 99%CI: 1.02–1.06) or ADHD medications (aPR 1.14; 99%CI: 1.07–1.2), while they were less likely than females to receive antidepressants (aPR 0.83; 99%CI: 0.82–0.85) or anxiolytics (aPR 0.86; 99%CI: 0.84–0.88). No statistically significant differences existed in the prevalence of mood stabilizer use between males and females.

**Table 2:** Association between sex and likelihood of being exposed to psychiatric medications in the year after the first diagnosis of cluster B personality disorder

	Male *N*^#^ (%)	Female *N*^#^ (%)		PR (99% CI)	*P*-value	aPR* (99% CI)	*P*-value
**Psychiatric medications**							
**Antipsychotics**	17,705 (47.4)	21,925 (43.5)	**Male** vs. **Female**	1.09 (1.07–1.11)	< 0.0001	1.04 (1.02–1.06)	< 0.0001
**Antidepressants**	17,395 (46.6)	30,575 (60.6)		0.77 (0.76–0.78)	< 0.0001	0.83 (0.82–0.85)	< 0.0001
**Anxiolytics**	14,000 (37.5)	22,605 (44.8)		0.84 (0.82–0.85)	< 0.0001	0.86 (0.84–0.88)	< 0.0001
**Mood stabilizers**	6355 (17.0)	8575 (17.0)		1.00 (0.96–1.04)	0.9649	0.98 (0.95–1.02)	0.2785
**ADHD medications**	2375 (6.4)	2800 (5.6)		1.15 (1.07–1.23)	< 0.0001	1.14 (1.07–1.22)	< 0.0001

ADHD: attention-deficit/hyperactivity disorder; CI: confidence interval; ^#^randomly rounded to 0/5; *Adjusted for age, material and social deprivation, the year of Cluster B PD diagnosis, and psychiatric comorbidities in the 5 years before Cluster B PD diagnosis; PR: prevalence ratio

Supplemental Fig. 2 showed trends in the use of psychotropic medication classes over the study period. After diagnosis, females were more likely to receive psychotropic medications, with the proportion of users ranging between 78.9% and 83.7% during the study period, compared to 72.8% and 76.8% among males (Supplemental Fig. 2, letter A). In general, trends over time were slightly similar for both males and females, showing steady use in the last two decades, with females being more likely to use psychotropic medications. Particularly, they tended to use more antidepressants and anxiolytics, while males used antipsychotics. The proportion of users was similar among both sexes for ADHD medications and mood stabilizers, with a higher proportion of users recorded in the year after PD diagnosis than before for all the years in the study period. Trends over time showed an important decrease in the use of anxiolytics and mood stabilizers and an increase in ADHD medications and antipsychotics, while antidepressants remained stable.

## Discussion

To the best of our knowledge, this is the first study to leverage a large dataset of Cluster B PD individuals to elucidate the nuanced variations in psychopharmacological treatment across sex and age, shedding light on critical epidemiological patterns. Our findings revealed three key insights: (i) a noticeable fluctuation in prescription patterns surrounding the point of diagnosis; (ii) a trend towards higher medication use among females, with the exception of antipsychotics; and (iii) a significant variability in prescribing practices according to both age and classes of psychotropic medications.

The data indicated a surge in medication prescriptions nearing the time of diagnosis for both sexes, followed by a decline post-diagnosis that nonetheless failed to revert to pre-diagnosis levels, suggesting complex dynamics in treatment initiation and continuation. Several hypotheses have been proposed to explain this situation, including the emergence of new comorbidities, the sudden decompensation of an individual’s health due to an adverse social situation, the limited access to affordable psychotherapy, or the dynamics in patient-doctor relation, which can affect the patient’s tolerance of symptoms and their request for medication. It seems that during periods of crisis, clinicians are more prone to identify Cluster B PD in their patients. Thus, the diagnosis may remain hidden [29] until the clinician observes certain additional behaviours commonly associated with the diagnosis, such as suicidal tendencies or impulsive actions. This should lead to two clinical reflections. Firstly, it would be beneficial to screen for PD in groups with affective disorders or ADHD who are taking psychiatric medications before adding any new drug. This proactive approach could help to identify and treat PD, possibly with psychotherapy, before a crisis occurs. Secondly, after the “diagnostic crisis,” it is recommended that clinicians initiate deprescribing, as prescriptions decreased but did not revert to previous levels, despite clinical guidelines cautioning against these prescriptions [30, 31].

The tendency for female patients to be prescribed more medications than their male counterparts echoes broader international trends, pointing towards gender-based disparities in healthcare access, as women generally receive more medical attention and treatment strategies within psychiatric settings [24, 32–34]. The reasons for this could stem from biological or psychological distinctions and social determinants. It is widely recognized that there are notable distinctions between women and men with Cluster B PD, such as the number of lost years of life compared with the general population [2]. Specifically, our previous research has shown that, at age 20, female patients with Cluster B PD have a life expectancy reduced by up to nine years compared to the general population, while male patients with Cluster B PD may experience a reduction of up to 13 years [2]. The presence of comorbidities differed significantly between sexes, which may account for using distinct medication approaches. Women may experience more affective, anxiety, and eating disorders, possibly resulting in a higher likelihood of being prescribed antidepressants [21]. Meanwhile, men may exhibit higher rates of antisocial personality disorder, substance use disorders, explosive behaviours, and aggressiveness, potentially leading to increased prescription of antipsychotic medications [6, 21, 35]. Moreover, the prescription of psychotropic drugs may vary depending on sex or gender, even when treating the same illness. This is partially due to the differing effect size observed between sexes [36, 37]. This may not address the gender bias that plagues the healthcare system. Due to various social and demographic factors, there may be an overemphasis on medicalizing women and a corresponding lack of attention given to men [38]. Paradoxically, this could result in advocating for a decrease in prescription rates among females while simultaneously increasing awareness of treatment options for males.

The pronounced age-related variations in prescription trends across sexes underscore the profound impact of age as a determinant of psychiatric care, warranting deeper exploration. For instance, antipsychotic medication was more frequently prescribed for individuals of both sexes between the ages of 50 and 64 years. This could be partially explained by the possible use of some antipsychotics, such as quetiapine, for insomnia, a condition that is highly prevalent in this age group [39, 40]. Indeed, in a sensitivity analysis, removing prescriptions for low doses of quetiapine (50 mg or less), which are typically used off-label for sleeping purposes rather than psychiatric disorders, resulted in a marked reduction in the overall count of antipsychotic usage, as illustrated in Supplemental Fig. 1 [39]. Despite this reduction, the use of antipsychotics after the diagnosis remained higher than before the diagnosis for all groups, albeit to a lesser extent in women aged 50–64. These results need further investigation and a specific focus on antipsychotics that goes beyond the purpose of the current paper.

Antidepressants and anxiolytics were more commonly prescribed to men between 50 and 64, while women over 65 received a higher proportion of these drugs. Finally, with regard to ADHD medications, the trend was reversed, with a greater frequency of prescriptions observed among younger individuals that decreased over time. There has been a discernible rise in the prescription of ADHD medication to young individuals, and this trend can be attributed to different discernable factors. On the one hand, the diagnosis has not been recognized enough in the past [41–44]. On the other hand, young people may require enhanced focus for their studies, which could contribute to the greater need for ADHD drugs in this population. The increasing use of ADHD medications over time may also reflect the growing evidence of their effectiveness in reducing hospitalizations, injuries and mortality in ADHD patients [45, 46], and also among patients with BPD [47]. Comprehending the peak in antidepressant and antipsychotic prescriptions and the sex-based disparities therein poses a greater challenge. Above all, it is important to remember that prescriptions may not always be associated with an officially approved indication, as medication can be used off-label [48]. By considering this perspective, we can speculate that in males with Cluster B PDs, the highest occurrence of behavioural symptoms (such as impulsive behaviours) and internal symptoms (such as anxiety and depression) is typically observed between the ages of 50 and 64. Following this period, impulsive symptoms may tend to decrease. Conversely, among females, the peak of behavioural problems precedes that of internal symptoms. It is important to note that while BPD symptoms generally diminish over time [13, 14], the overall progression of the entire Cluster B PD may not follow the same pattern.

There was no discernible difference in trends over time between the sexes. The most prevalent medication category utilized by both males and females was antidepressants, in line with the fact that depression and anxiety were the most common comorbid conditions. Indeed, the main indications for antidepressant prescriptions are depressive [49] and anxiety disorders [50]. Indeed, anxiolytic use decreased over time, partly because of guidelines recommending newer antidepressants instead of anxiolytics for both depressive and anxiety symptoms [50]. The decline in the use of anxiolytics has also been reported in other studies from Quebec [51, 52]. However, there was no increase in the use of antidepressants during the study period to compensate for the decrease in anxiolytic use. Antipsychotic use increased only slightly during the study period. However, there was a noticeable increase in usage in the year following a diagnosis of PD, suggesting higher compliance with guidelines in the latest years [17, 18]. Nevertheless, low doses of antipsychotics can be used successfully to control emotional crises, especially in BPD, and a subclass of patients may present true psychotic symptoms. However, a possible inappropriate prescription of antipsychotics once the PD is diagnosed can still be present. Finally, prescriptions of ADHD medications showed a constant rise over the study period, corresponding to the increasing number of ADHD diagnoses over the past few years, regardless of the presence of Cluster B PD [43, 44]. The data also shows that an increasing number of females are taking ADHD medication, which is a possible indicator of a positive trend in identifying and treating this condition among girls and young women.

It is important to acknowledge that our study had some limitations. First, we used the information in the QICDSS database to categorize individuals based on sex. Even if it is theoretically possible for individuals to ask for a modification in the information about sex in the database, we believe that the frequency should be low during the period considered, but we do not have this information. We are not aware of the frequency of this occurrence. This means that our results do not apply to gender differences but rather to sex differences. Moreover, we could not access all the necessary information about medication use for every patient but only for those covered by the public drug plan. Therefore, our findings may not apply to all individuals with Cluster B PDs. This is because people with private insurance tend to have higher socioeconomic status and possibly less severe cases, as they can work and access and maintain employer-provided private drug insurance. Moreover, they have access to private health insurance covering psychotherapy, which may not be affordable for those who are more disadvantaged. Additionally, we used the diagnostic ICD-9 and ICD-10 codes recorded in the QICDSS database to identify individuals with Cluster B personality disorders. Despite the choice of such codes being based on a consensus among expert clinicians working with PD patients in Quebec, some individuals may have been wrongly assigned these codes or have been misdiagnosed. Moreover, it is important to note the absence of specific ICD codes exclusively for Cluster B PDs, which led to the adoption of a general set of codes deemed appropriate by these clinicians. This methodological choice lacks a formally validated case definition, which could lead to diagnostic misclassifications. As a result, individuals with Cluster A or C personality disorders may have been mistakenly classified as having Cluster B disorders. Considering that we employed medication claims as an estimate for medication usage, it is also possible that we may have overestimated the proportion of individuals taking psychotropic medications. Nonetheless, our findings align with previous studies conducted in various countries and contexts, indicating a high prevalence of medication use among individuals with PDs. It is also important to acknowledge that the SISMACQ database, while extensive, does not encompass all the potential confounding variables that could influence the association between sex and psychotropic prescribing patterns, such as severity of symptoms or patient-reported outcomes.

Despite these limitations, our study was bolstered by the large and diverse sample size. Indeed, it used a large cohort of Cluster B PD individuals throughout the Canadian province of Quebec. Furthermore, we had access to almost two decades of data, allowing us to discern patterns and trends in psychotropic medication use and compare them according to sex and age groups. Finally, while this study provides comprehensive insights into psychotropic medication use across a large population, detailed analyses concerning specific medication dosages and their off-label use were beyond the scope of this initial investigation. Future studies could benefit from exploring these aspects to refine the understanding of treatment patterns in cluster B patients, particularly for medications frequently used off-label (e.g., quetiapine).

## Conclusions

The clear differences observed between various age and sex groups suggest significant disparities in prescription drug usage, warranting further investigation. The observed differences in prescription drug usage may be partially attributable to the differences in psychiatric conditions and comorbidities between males and females, different age groups, and changes in clinical practice over time. Nevertheless, it is also possible that social determinants, as seen in other fields, may play a role in shaping appropriate or inappropriate treatment for patients. Thus, our findings provide a valuable starting point for future research in this field that may drive clinical awareness and future decisions in treating these patients.

## Electronic supplementary material

Below is the link to the electronic supplementary material.

Supplementary Material 1

## Data Availability

No permission is granted to use the Quebec Integrated Chronic Diseases Surveillance System (QICDSS) data.
