# Supplementary material for: Cluster B personality disorders and psychotropic medications: a focused analysis of trends and patterns across sex and age groups
Source: Soc Psychiatry Psychiatr Epidemiol. 2024 Sep 17;60(6):1499–509. doi: 10.1007/s00127-024-02768-1 (PMC12162776; doi:10.1007/s00127-024-02768-1)
Supplement: Supplementary file 1 — Supplementary Material 1 [file 127_2024_2768_MOESM1_ESM.docx]

**Supplemental Figure 1. Proportion of individuals exposed to antipsychotic medications, excluding and including low-dose Quetiapine (100 mg or less) in the 12 months before and after the Cluster B personality disorder diagnosis, by month, according to sex and age groups**

**Supplemental Figure 2. Proportion of individuals exposed to psychotropic classes in the year before (dotted lines) and after (solid lines) Cluster B personality disorder (PD) diagnosis, by sex and the year of PD diagnosis**

**Supplemental Table 1. Psychotropic medications with their common denomination codes and AHFS classification**

| **Group** | **Sub-group** | **Medications** | **AHFS classification** | **Common denomination codes** |
| --- | --- | --- | --- | --- |
| **Antipsychotics** | Typical | Chlopromazine | 28:16.08.24 | 1924 |
|  |  | Flupenthixol |  | 41863  43202 |
|  |  | Fluphenazine | 28:16.08.24 | 4056  4069  34284 |
|  |  | Haloperidol | 28:16.08.08 | 4394  43540  43826  46292 |
|  |  | Loxapine | 28:16.08.92 | 34219  37612  40745 |
|  |  | Methotrimeprazine |  | 6045 |
|  |  | Perphenazine | 28:16.08.24 | 7176  46011  (In combination with amitryptiline) |
|  |  | Pimozide | 28:16.08.92 | 33465 |
|  |  | Pipotiazine |  | 41707 |
|  |  | Prochlorperazine | 28:16.08.24 | 45458  45528  8125 |
|  |  | Thioridazine | 28:16.08.24 | 9594 |
|  |  | Thioproperazine |  | 9568 |
|  |  | Trifluoperazine | 28:16.08.24 | 9802  34440  46108  (In combination with isopropamide) |
|  |  | Zuclopenthixol |  | 47136  47137  47138 |
|  | Atypical | Asenapine | 28:16.08.04 | 47921 |
|  |  | Aripiprazole | 28:16.08.04 | 47801 |
|  |  | Brexpiprazole | 28:16.08.04 | 48153 |
|  |  | Clozapine | 28:16.08.04 | 45580 |
|  |  | Lurasidone | 28:16.08.04 | 47939 |
|  |  | Olanzapine | 28:16.08.04 | 46318  47197 |
|  |  | Paliperidone | 28:16.08.04 | 47708  47861 |
|  |  | Quetiapine | 28:16.08.04 | 47267 |
|  |  | Risperidone | 28:16.08.04 | 46156  47052 |
|  |  | Ziprazidone | 28:16.08.04 | 47717 |
| **Antidepressants - Those mostly used for depression and anxiety disorders** | SSRIs | Citalopram | 28:16.04.20 | 46543  47317 |
|  |  | Escitalopram | 28:16.04.20 | 47553  47971 |
|  |  | Fluoxetine | 28:16.04.20 | 45504 |
|  |  | Fluvoxamine | 28:16.04.20 | 45633 |
|  |  | Paroxetine | 28:16.04.20 | 47061 |
|  |  | Sertraline | 28:16.04.20 | 45630 |
|  |  | Vilazodone | 28:16.04.20 | 48227 |
|  | SNRIs | Desvenlafaxine | 28:16.04.16 | 47770 |
|  |  | Duloxetine | 28:16.04.16 | 47714 |
|  |  | Levomilnacipran | 28:16.04.16 | 48075 |
|  |  | Venlafaxine | 28:16.04.16 | 46244  47118 |
|  | NDRIs | Bupropion | 28:16.04.92 | 46435  47285  48205  (In combination with naltrexone) |
|  | NaSSAs | Mirtazapine | 28:16.04.92 | 46744  47408 |
|  | MAOI | Phenelzine | 28:16.04.12 | 7280 |
|  |  | Tranylcypromine | 28:16.04.12 | 9698 |
|  | RIMA | Moclobemide |  | 46427  47005 |
|  | Serotonin modulator | Vortioxetine | 28:16.04.24 | 48038 |
|  |  | Trazodone | 28:16.04.24 | 43137 |
| **Other antidepressants - Those mostly used for other indications than depression or anxiety disorders** | Tricyclics | Amitryptiline | 28:16.04.28 | 429  46011  (Combination with perphenazine) |
|  |  | Clomipramine | 28:16.04.28 | 14781 |
|  |  | Desipramine | 28:16.04.28 | 2522 |
|  |  | Doxepin | 28:16.04.28 | 3198 |
|  |  | Imipramine | 28:16.04.28 | 4784 |
|  |  | Nortriptyline | 28:16.04.28 | 6578 |
|  |  | Trimipramine | 28:16.04.28 | 9906 |
| **Mood stabilizers (other than antipsychotics and other medications included in other classes)** | | Carbamazepine | 28:12.92 | 1404  10270 |
|  |  | Gabapentin | 28:12.92 | 46229  47100 |
|  |  | Lamotrigine | 28:12.92 | 47110  46248 |
|  |  | Lithium | 28:28 | 47071  47237  47589  5330 |
|  |  | Oxcarbazepine | 28:12.92 | 46805  47430 |
|  |  | Topiramate | 28:12.92 | 46359  47229 |
|  |  | Valproic acid | 28:12.92 | 38951  39393  44073 |
| **Anxiolytics** | Benzodiazepines | Alprazolam | 28:12.08 | 43501 |
|  |  | Bromazepam | 28:12.08 | 43488 |
|  |  | Chlordiazepoxide | 28:12.08 | 1807 |
|  |  | Clobazam | 28:12.08 | 45591 |
|  |  | Clonazepam | 28:12.08 | 37872 |
|  |  | Clorazepate | 28:12.08 | 14768 |
|  |  | Diazepam | 28:12.08 | 2717 |
|  |  | Flurazepam | 28:12.08 | 4095 |
|  |  | Lorazepam | 28:12.08 | 37950 |
|  |  | Nitrazepam | 28:12.08 | 42045 |
|  |  | Oxazepam | 28:12.08 | 6786 |
|  |  | Temazepam | 28:12.08 | 41590 |
|  |  | Triazolam | 28:12.08 | 39029 |
|  | Others | Buspirone | 28:24.92 | 45609 |
| **ADHD medications** | | Amphetamine | 28:20.04 | 507  47601  48001 |
|  |  | Amphetamine/  Dexamphetamine | 28:20.04 | 47486 |
|  |  | Atomoxetine | 28:92 | 47547 |
|  |  | Dexamphetamine | 28:20.04 | 2626 |
|  |  | Lisdexamfetamine | 28:20.04 | 47818  48000 |
|  |  | Methylphenidate | 28:20.32 | 48003  39302 |
|  |  | Guanfacine | 24:08.16 | 47979 |

5-HT2: 5-Hydroxytryptamine 2 subtype; ADHD: Attention-deficit hyperactivity disorder; AHFS: American hospital formulary service; MAOI: Monoamine oxidase inhibitors; NaSSAs: Noradrenergic and specific serotonergic antidepressants; NDRIs: Norepinephrine and Dopamine Reuptake Inhibitors; SNRIs: Serotonin and norepinephrine reuptake inhibitors; SSRIs: Selective serotonin reuptake inhibitors.
